# Long non‐coding RNAs as therapeutic targets in head and neck squamous cell carcinoma and clinical application

**DOI:** 10.1002/2211-5463.70042

**Published:** 2025-04-15

**Authors:** Ellen T. Tran, Ruchi A. Patel, Amogh Chariyamane, Ratna B. Ray

**Affiliations:** ^1^ Department of Pathology Saint Louis University MO USA

**Keywords:** gene regulation, head and neck cancer, long non‐coding RNA, RNA‐binding protein, therapeutic target

## Abstract

Head and neck squamous cell carcinoma (HNSCC) is a major global health burden, often associated with poor prognosis and limited therapeutic options. Long non‐coding RNAs (lncRNAs), a diverse group of non‐coding RNA molecules > 200 nucleotides in length, have emerged as critical regulators in the pathogenesis of HNSCC. This review summarizes the mechanisms through which certain lncRNAs regulate chromatin modification, mRNA splicing, and interactions with RNA‐binding proteins and contribute to the development and progression of HNSCC. Interaction of lncRNAs with key oncogenic pathways, such as PI3K/AKT and Wnt/β‐catenin, highlights their importance in tumor progression. The role of lncRNAs, such as ELDR, MALAT1, NEAT1, HOTAIR, and UCA1, which promote cell proliferation, metastasis, immune evasion, and therapy resistance is discussed. Moreover, several lncRNAs are being evaluated in clinical trials for their potential as biomarkers, reflecting their clinical significance. We further address the challenges and opportunities for targeting lncRNA therapeutically, highlighting the promise of lncRNA‐based interventions for personalized cancer treatment. Gaining insight into the function of lncRNAs in HNSCC could pave the way for novel therapeutic strategies to potentially improve patient outcomes.

## Graphical abstract

Long non‐coding RNAs (lncRNAs) occupy an abundant fraction of the eukaryotic transcriptome and an emerging area in cancer research. Regulation by lncRNAs is based on their subcellular localization in HNSCC. This cartoon shows the various functions of lncRNAs in HNSCC discussed in this review.

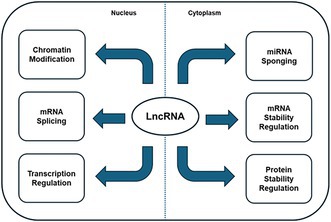

**Abbreviations**
ANLN: anillin actin binding protein
ANRIL: antisense non‐coding RNA in the INK4 locus
ATM‐CHK2: ataxia telangiectasia mutated‐checkpoint kinase 2
AURKA: Aurora Kinase A
CAGE: cap analysis gene expression
ceRNA: competing endogenous RNA
CSC: cancer stem cell
CTCF: CCCTC‐binding factor
DNMT1: DNA methyltransferase 1
EGFR: epidermal growth factor receptor
EGFR‐AS1: epidermal growth factor receptor antisense RNA 1
ELDR: EGFR long non‐coding downstream RNA
EMT: epithelial‐to‐mesenchymal transition
ESCC: esophageal squamous cell carcinoma
EZH2: enhancer of zeste homolog 2
FIRRE: functional intergenic repeat RNA element
FOXM1: forkhead box M1
FZD3: frizzled class receptor 3
GCN5: general control non‐depressible 5
HNRNPK: heterogeneous nuclear ribonucleoprotein K
HNSCC: head and neck squamous cell carcinoma
HOTAIR: HOX transcript antisense intergenic RNA
HOTTIP: HOXA transcript at the distal tip
HPV: human papillomavirus
HSCC: hypopharyngeal squamous cell carcinoma
HuR: human antigen R
IGF2BP2: insulin‐like growth factor 2 mRNA‐binding protein 2
ILF3: interleukin enhancer binding factor 3
JAK/STAT3: Janus kinase/signal transducer and activator of transcription 3 pathway
LINC: long intergenic non‐coding RNA
LINRIS: long intergenic non‐coding RNA for IGF2BP2 stability
lncRNA: long non‐coding RNA
LSCC: laryngeal squamous cell carcinoma
LSD1: lysine‐specific demethylase 1
m6A: N6‐methyladenosine
MALAT1: metastasis‐associated lung adenocarcinoma transcript 1
MEG3: maternally expressed gene 3
MEK/ERK: mitogen‐activated protein kinase/extracellular signal‐regulated kinase pathway
METTL14: methyltransferase‐like 14
miRNA: microRNA
mRNA: messenger RNA
MTFR1: mitochondrial fission regulator 1
ncRNA: non‐coding RNA
NEAT1: nuclear‐enriched abundant transcript 1
NEAT2: nuclear‐enriched abundant transcript 2
NF‐κB: nuclear factor kappa B
NPC: nasopharyngeal carcinoma
NSCLC: non‐small cell lung cancer
OSCC: oral squamous cell carcinoma
OTSCC: oral tongue squamous cell carcinoma
PABPN1: polyadenylate‐binding protein 1
PCAT‐1: prostate cancer‐associated transcript 1
PD1: programmed cell death protein 1
PI3K/AKT: phosphoinositide 3‐kinase/protein kinase B pathway
PRC2: polycomb repressive complex 2
PRMT5: protein arginine methyltransferase 5
PTENP1: phosphatase and tensin homolog pseudogene 1
PVT1: plasmacytoma variant translocation 1
RBP: RNA‐binding protein
rRNA: ribosomal RNA
SNHG1: small nucleolar RNA host gene 1
snoRNA: small nucleolar RNA
snRNA: small nuclear RNA
SOX4: SRY‐box transcription factor 4
SR: serine/arginine‐rich
TCGA: The Cancer Genome Atlas
TGF‐β: transforming growth factor beta
TINCR: terminal differentiation‐induced non‐coding RNA
TLR5: toll‐like receptor 5
tRNA: transfer RNA
TSCC: tongue squamous cell carcinoma
TUG1: taurine upregulated gene 1
UCA1: urothelial carcinoma associated 1
VEGF: vascular endothelial growth factor
XIST: X‐inactive specific transcript

## General description of lncRNAs

The classical central dogma of molecular biology states that DNA encodes RNA, which encodes proteins. However, genomic analyses have demonstrated a startling fact: < 5% of the human genome encodes proteins. The remaining vast majority of the genome was once labeled as “junk” with no functional importance. However, research over the recent decades has challenged and outdated this notion [1], thereby revolutionizing our understanding of the central dogma of molecular biology. It is now well established that significant portions of DNA encode non‐coding RNAs (ncRNAs), which play important roles in various physiological functions, such as gene regulation and disease progression. Both transfer RNA (tRNA) and ribosomal RNA (rRNA) are crucial players in the process of translating messenger RNA (mRNA) transcripts within ribosomes [2], while small nuclear RNA (snRNA) aids in the splicing of mRNA primary transcripts [3], small nucleolar RNA (snoRNA) contributes towards rRNA processing [4], and microRNA (miRNA) works to repress expression of target genes [5]. Several other classes of ncRNAs also exist and are constantly being discovered. Research on ncRNAs is becoming increasingly impactful. In fact, the 2024 Nobel Prize in Medicine or Physiology was awarded for research on microRNAs. One of the most diverse groups of ncRNAs is lncRNA, which has been the center of research in recent years. The lncRNAs are defined as RNA transcripts that are not translated into proteins and are composed of at least 200 nucleotides [6].

Several reports have revealed that lncRNAs are major components of the human genome. Nearly 28 000 human lncRNA genes were identified in a 2017 study utilizing FANTOM5 cap analysis of gene expression (CAGE) data [7], while approximately 95 000 human lncRNA genes were identified in 2021 using analysis with a different repository called NONCODE [8]. According to current databases, there are fewer than 20 000 protein‐coding genes in the human genome [9]. The continuous discovery of new lncRNAs adds to the uncertainty surrounding the precise number of lncRNA genes, but it is evident that lncRNAs are extremely common, play important physiological roles, and defy the long‐held belief that they are merely byproducts of background transcription “noise”. The lncRNAs are remarkable for their presence in diverse species and forms of life. They exhibit limited conservation among these species, a unique characteristic that sets them apart from many other RNA types known for their higher conservation levels [10]. Many lncRNAs exhibit characteristics similar to mRNAs, having multiple exons and a 5′ cap and 3′ poly‐A tail. However, lncRNAs differ from protein‐coding genes as they are highly tissue‐specific and are often expressed at low levels, particularly in intricate tissues [11].

The most distinctive feature of lncRNAs is their heterogeneity. They have varying compositions and contribute towards a wide array of biological processes. This heterogeneity has led to multiple different attempts to categorize lncRNAs. Ma et al. classified the lncRNAs according to four different characteristics: genomic location and context, exerted effect on DNA sequences, mechanism of functioning, and targeting mechanism [12]. Within genomic location and context, lncRNAs can fall into the categories of being either intergenic (transcribed from regions between genes) or intronic (transcribed from the introns of protein‐coding genes), and either sense or antisense depending on which strand of DNA the lncRNA is transcribed from. LncRNAs can be understood in terms of their effect on DNA sequences as being either cis‐acting or trans‐acting, depending on whether they influence proximally located or distally located genes, respectively. Types of functioning mechanisms for lncRNAs include transcriptional regulation, post‐transcriptional regulation, telomere replication, and RNA interference. Some lncRNAs work by binding and removing a protein target from the matrix, whereas others work by controlling the localization of ribonucleoprotein complexes.

These vastly diverse properties and roles of lncRNAs provide an explanation for their diverse association with several human diseases such as myocardial infarction [13, 14, 15], Huntington's disease, Alzheimer's, amyotrophic lateral sclerosis, and Parkinson's disease [16], and a range of human cancers, such as breast, colon, brain, lung, liver, bladder, and head and neck [17]. This review will provide the role of lncRNAs in relation to head and neck cancer, particularly their potential as a therapeutic target and their clinical application.

## Head and neck cancer

HNSCC is the seventh most common cancer in the world, accounting for an estimated 890 000 new cases and 450 000 deaths per year [18]. By the year 2030, it is estimated that over 1 million more cases of HNSCC will occur annually [19], indicating the rising incidence of the cancer. HNSCC refers to a group of biologically similar cancers that start in the lip, tongue, oral cavity (mouth), nasal cavity (inside the nose), paranasal sinuses, pharynx, and larynx. HNSCC prevalence differs based on global region, with cancers of the tonsils and pharynx being highest in Western Europe, cancers of the larynx being highest in the Caribbean region, and cancers of the nasopharynx heavily affecting Northern Africa and Asia [18, 20]. HNSCCs are highly heterogeneous and contain many genetic alterations that make them refractory to specific targeted drugs.

The risk factors for HNSCC are mostly known. Alcohol and tobacco use are strongly associated with this cancer. There is evidence that over 30% of pharyngeal cancers and over 20% of laryngeal cancers are attributable to alcohol use [21]. The amount of alcohol and the timespan of drinking alcohol play a role in determining the risk. The exact mechanism through which alcohol causes HNSCC has not been fully elucidated; however, one possible avenue may be through DNA damage caused by the products of alcohol metabolism. Along with alcohol, tobacco (smoked or chewed) use is a significant driving factor for head and neck cancers. In fact, the chances of developing HNSCC in those who smoke are 2.13 times higher than among non‐smokers, and it is unproven whether cessation from smoking can fully eliminate the increase in risk [22]. Although both alcohol and tobacco are independent risk factors, they have an even greater effect on increasing the risk of developing HNSCC when used in tandem, with the effect most pronounced in males and in older populations [23]. Human papillomavirus (HPV) is another significant causal factor for the development of HNSCC. This double‐stranded DNA virus can infect epithelial cells in the head and neck region, and it is responsible for up to 60% of the cancers of the oropharyngeal subtype, with the HPV16 strand being the most common responsible agent [24]. However, HPV infection is predicted to decline in the future due to successful vaccination campaigns and better prognosis [18].

There are various treatment options for HNSCC, including surgical removal, radiation therapy, chemotherapy, targeted therapy, and immunotherapy. One of the most effective ways to avoid this cancer mortality in patients is to surgically remove the tumor from its local primary site [25]. Radiation therapy is often used to kill cancerous cells, but there are a variety of acute and delayed adverse effects associated with HNSCC radiation therapy. Acute side effects of radiation for HNSCC can include mucositis, dermatitis, malnutrition, dehydration, dry mouth, and pain when swallowing [26]. Delayed effects can include radiation necrosis, dental caries, hypothyroidism, hyperparathyroidism, fibrosis, damage to the eyes and brain, and secondary cancer [27]. Chemotherapy has been extensively researched in relation to head and neck cancer, with meta‐analysis showcasing the increased survivability from chemotherapy, and greater benefit from concomitant chemoradiotherapy compared to induction or adjuvant chemotherapy [28]. Targeted therapies have promising effects in fighting against HNSCC, with EGFR signaling being the central target for this cancer, and VEGF and MEK/ERK signaling serving as other classic targets [29]. Research in immunotherapy for HNSCC is also rapidly growing and leading to promising results, such as the use of the anti‐PD1 antibody (pembrolizumab) holds a good promise [30].

## Mechanisms of lncRNA‐mediated regulation of HNSCC pathogenesis

### Gene regulation and chromatin modification

One of the most studied mechanisms is epigenetic regulation, where lncRNAs influence the structure and function of chromatin—the complex of DNA and proteins that makes up chromosomes. The lncRNAs can exert their physiological effects through regulating genes via chromatin modification. Chromatin is DNA that has been condensed within the nucleus of a cell, organized in a “beads on a string” model. In this model, histone proteins, specifically eight of them consisting of two copies each of H2A and H2B and H3 and H4, bind to and are wrapped around by DNA, forming a nucleosome. DNA can either be inaccessible to transcription in a tightly condensed heterochromatin form or accessible to transcription in a more loosely condensed euchromatin form. The lncRNAs can recruit chromatin remodeling complexes to specific areas of the genome, compete with the binding of these complexes, inhibit their expression, alter their ubiquitination, or control the transcription of their protein subunits [31]. For example, lncRNA HOTAIR interacts with Polycomb Repressive Complex 2 (PRC2), a histone‐modifying complex that trimethylates the histone H3 on lysine 27, and silences genes [32]. LncRNA RASAL2‐AS1 interacts with METTL14, a methyltransferase that adds m6A modifications (a form of RNA methylation) to mRNA and affects mRNA stability and protein production in HNSCC cells [33]. The lncRNA ANRIL has been shown to modulate chromatin structure through its interaction with PRC2, regulating cell cycle genes in various cancers, including HNSCC [34]. The tumor suppressor silencing function of lncRNAs often involves their interaction with chromatin‐modifying proteins, leading to transcriptional repression. For example, ANRIL silences key tumor suppressor genes in various cancers, including in HNSCC and promotes tumor growth [35]. On the other hand, lncRNA MX1‐215 forms complex with GCN5 and functions as a tumor suppressor in HNSCC, inhibiting cell proliferation and metastasis. In a recent study, Song et al. reported that the role of super enhancer‐associated lncRNAs in reprogramming the transcriptional landscape of HNSCC [36]. This scaffolding role of lncRNAs allows them to influence transcriptional repression in HNSCC, contributing to the silencing of tumor suppressor genes and the promotion of oncogenesis.

### mRNA splicing

Another key function of lncRNAs is their involvement in mRNA splicing, a process where pre‐mRNA is modified to remove introns and join exons together, creating mature mRNA ready to translate into proteins. LncRNAs can interact with components of the splicing machinery, influencing alternative splicing events that can result in different protein isoforms. For example, MALAT1 plays a role in the regulation of splicing factors such as SR proteins (serine/arginine‐rich proteins) and has been shown to alter the splicing patterns of various mRNAs, thereby impacting gene expression profiles that drive tumor cell proliferation and migration [37]. This regulatory role is crucial for processes like epithelial‐to‐mesenchymal transition (EMT), where alternative splicing can promote a shift from an epithelial phenotype to a more invasive mesenchymal state, thus enhancing metastasis. MALAT1 regulates the alternative splicing of ANLN, a protein involved in cytoskeletal dynamics, in HNSCC. The altered splicing contributes to the production of different ANLN isoforms, which have distinct roles in promoting tumor proliferation and invasion in HNSCC cells [38].

### Sequestration of miRNA

LncRNAs also sequester miRNAs and prevent miRNAs from binding to their target mRNAs. This process, known as the ceRNA (competing endogenous RNA) network, allows lncRNAs to act as molecular sponges [39]. Earlier studies on PTENP1, a pseudogene‐derived lncRNA, laid the foundation for understanding how lncRNAs can influence the availability of miRNAs, thus indirectly regulating the expression of critical oncogenes and tumor suppressors [40]. For example, LINC00662 in HNSCC has been shown to sponge miR‐15b‐5p, thereby protecting oncogenic mRNAs from miRNA‐mediated degradation [41]. By sequestering miRNAs, LINC00662 ensures the continued expression of target genes that promote tumor cell proliferation and survival, thus facilitating cancer progression. By stabilizing mRNAs, lncRNA NEAT1 contributes to the sustained expression of genes that drive tumor cell proliferation and resistance to apoptosis [42]. In HNSCC, lncRNA PVT1 interacts with the c‐Myc mRNA, stabilizing it and thus enhancing the expression of this oncogene, which drives cell proliferation and tumor progression [43]. These interactions enable lncRNAs to modulate signaling pathways critical for HNSCC development and progression, and current research on the regulatory role of lncRNAs in various HNSCC signaling pathways will be discussed in the following sections of this review.

### Interaction with RNA‐binding proteins

LncRNAs physically associate with RNA‐binding proteins (RBPs) to exert their biological activities. RBPs have one or more domains that allow them to bind to and interact with RNA. By binding to RBPs, lncRNAs are able to regulate the post‐translational modification of these proteins. This can be done by various methods, such as by increasing their ubiquitination levels of RBPs and targeting them for degradation, by doing the opposite and decreasing RBP ubiquitination, or by inhibiting phosphorylation of RBPs [44]. For example, lncRNA Long Intergenic Non‐coding RNA for IGF2BP2 Stability (LINRIS) blocks K139 ubiquitination of Insulin‐like Growth Factor 2 mRNA‐Binding Protein 2 (IGF2BP2) and stabilizes IGF2BP2 protein to control aerobic glycolysis in colorectal cancer cells and promote this cancer growth [45]. NEAT1 is associated with RBPs like HNRNPK (heterogeneous nuclear ribonucleoprotein K) in various cancers including HNSCC and promotes tumor growth [46].

Interestingly, there is a two‐way path here: lncRNAs not only regulate RBPs but also are regulated themselves by RBPs. For example, the RBPs Human Antigen R (HuR) and Polyadenylate‐Binding Protein 1 (PABPN1) affect lncRNA stability and expression. HuR utilizes RNA recognition motifs to block signals for RNA degradation and stabilize lncRNAs such as NEAT1 and PABPN1 [47]. Conversely, some lncRNAs are degraded or sequestered by RBPs, which can impact their availability to carry out regulatory functions [48]. Additionally, the lncRNA and RBP binding can lead to tumor promotion. LINC00460 interacts with PRDX1, facilitating its nuclear translocation and promoting transcription of EMT‐related genes [49]. The lncRNA FIRRE associates with HuR and promotes hepatocyte growth [50]. Overall, the dysregulation of lncRNAs contributes to the aggressive behavior of cancers including HNSCC, which can make them potential targets for cancer therapy.

### Regulation of transcription

**Fig. 1:**
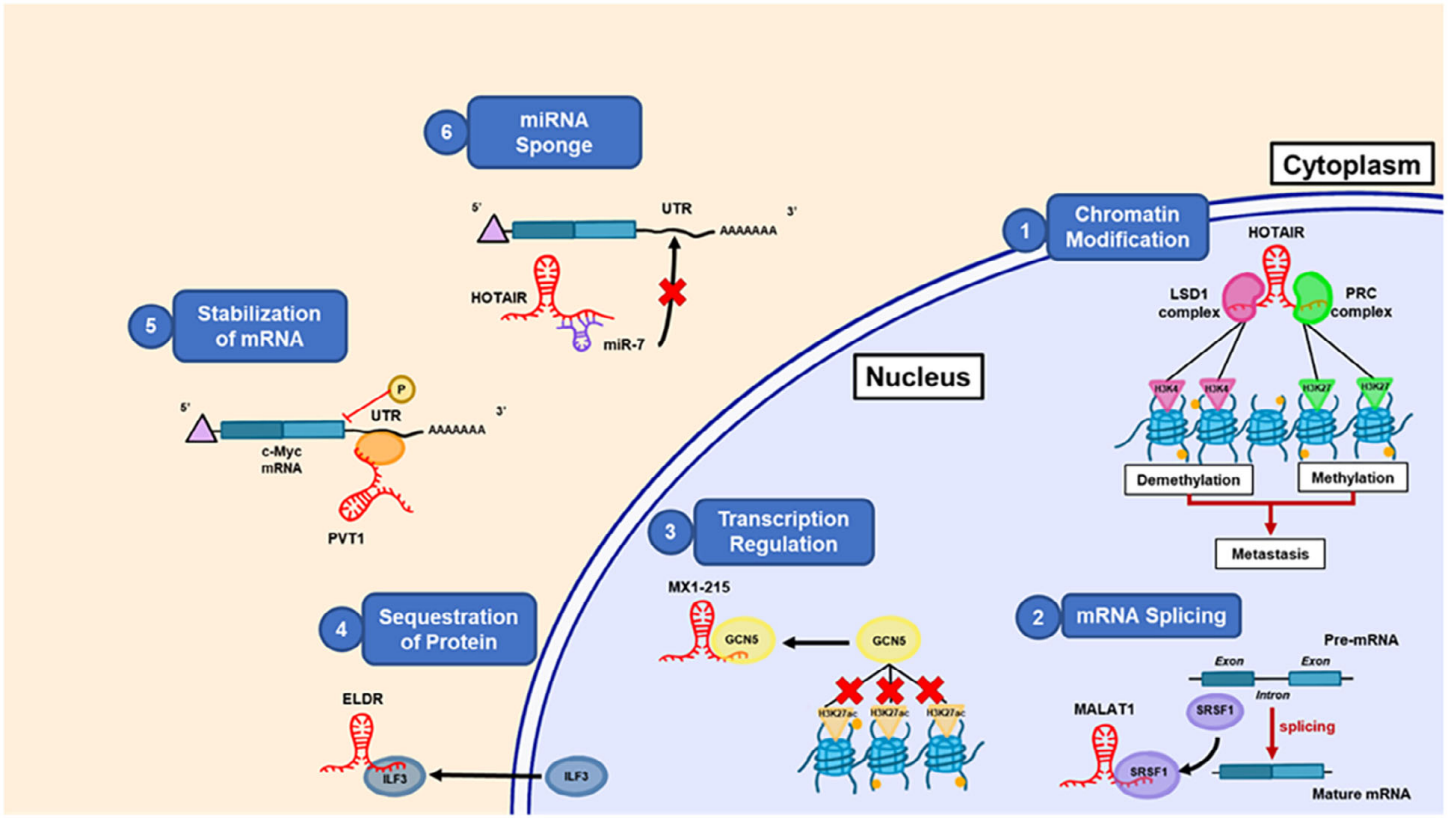
Molecular function of lncRNA with examples. 1–3: function of the lncRNAs in the nucleus, 4–6: function of the lncRNAs in the cytoplasm. 1: Epigenetic modification. HOTAIR interacts with the PRC or LSD1 complex and induces demethylation or methylation and promotes metastasis. 2: mRNA splicing: MALAT1 interacts with SRSF1 for mRNA splicing. 3: Transcription regulation: MX1‐215 binds GCN5 and modulates transcriptional activity. 4: Sequestration of protein: ELDR physically interacts with RNA‐binding protein ILF3 and sequesters ILF3 in the cytoplasm. 5: mRNA stabilization: PVT1 physically interacts and stabilizes mRNA by binding at an AU‐rich element in the 3′UTR. 6: Acting as sponge: HOTAIR acts as miRNA sponge and interacts with miR‐7 to form a complex, thus, miR‐7 cannot bind to the 3′ UTR of its target genes.

Transcription regulation is another common role that lncRNAs play. This is an expanding field of research, and the mechanisms through which lncRNAs can control transcription are only partially understood. The transcription regulation by lncRNAs can occur in a cis‐ (on the same DNA molecule) or trans‐ (on a different DNA molecule) acting manner. Furthermore, some lncRNAs have been found to contain binding sequences complementary to mRNA from distant genes, pointing towards in‐trans transcription regulation [51]. The lncSOX4 contributes to the development of liver cancer by recruiting the transcription factor STAT3 to the promoter of SOX4 [52]. In addition, lncRNAs can directly affect transcription by preventing RNA Polymerase II from binding to specific promoters [53]. Through these diverse mechanisms, such as involvement in mRNA splicing and acting as molecular scaffolds, regulating mRNA stability and sequestering miRNAs, lncRNAs play an integral role in the complex gene regulatory networks that drive the proliferation, survival, and invasiveness of HNSCC. These interactions not only help lncRNAs support tumor growth but also make them potential therapeutic targets for disrupting key oncogenic pathways. Figure 1 provides an overview of the key mechanisms of lncRNAs in HNSCC.

## Functional roles of lncRNAs in HNSCC development and progression

The lncRNAs play a pivotal role in regulating various aspects of HNSCC pathogenesis, including cell proliferation, survival, metastasis, treatment resistance, immune response, and angiogenesis [54, 55, 56, 57]. As discussed above, lncRNAs interact with mRNAs to regulate transcription, macromolecule synthesis, and immunological processes, including B and T cell signaling and TGF‐β receptor signaling [58, 59]. Studies as early as 2012 identified MALAT1 as a regulator of cell cycle progression, which promotes the growth of HNSCC cells by facilitating the transition between different stages of the cell cycle [37]. On the other hand, LINC00491 regulates growth through its interaction with the miR‐508‐3p/SATB1 axis, affecting the expression of genes that control cell proliferation in HNSCC [60].

LncRNAs also significantly contribute to metastasis and invasion. HOTAIR, for instance, has been extensively studied for its ability to promote EMT by silencing genes that inhibit metastasis, enabling cancer cells to detach and spread to other parts of the body [61]. LncRNA XIST (X‐inactive specific transcript) has been shown to interact with miR‐34a, a known tumor suppressor miRNA that targets genes involved in EMT [62]. By binding to miR‐34a, XIST prevents it from repressing target genes, allowing the expression of factors that promote EMT and metastasis. A recent study on LINC00662 further builds on this understanding by showing how lncRNAs can modulate miRNA networks, enhancing the migratory and invasive capabilities of HNSCC cells [41].

LncRNAs also modulate cancer stem cell (CSC)‐like properties, which are linked to tumor recurrence and therapy resistance. The lncRNA PCAT‐1 (Prostate Cancer‐Associated Transcript 1) has been shown to sustain the stemness of cancer cells, making them more resilient to conventional therapies [63]. Another study reported the role of PCAT6 with Notch signaling to maintain the self‐renewal capacity of CSCs in HNSCC [64]. FOXD2‐AS1 is involved in maintaining these properties through its interaction with STAT3, promoting self‐renewal and resistance to chemotherapy [65]. Similarly, the suppression of lincRNA‐p21 disrupts CSC characteristics and enhances sensitivity to therapy in HNSCC, making it a potential therapeutic target [66]. Another emerging role of lncRNAs in cancer biology is their regulation of angiogenesis, the process by which new blood vessels form to supply nutrients to tumors. MALAT1 has been implicated in promoting angiogenesis through upregulation of VEGF, although its specific role in HNSCC requires more targeted research [37]. The ability of lncRNAs to influence angiogenesis suggests their potential as targets to disrupt the blood supply to tumors.

Emerging evidence suggests that lncRNAs contribute to different signaling pathways in HNSCC by modulating PI3K/AKT, JAK/STAT3, TGF‐β/Smad, and Wnt/β‐catenin pathways [67, 68, 69]. The PI3K/AKT pathway is essential for regulating cell survival and proliferation, and the Wnt/β‐catenin signaling pathway is a critical regulator of cellular growth and proliferation, often implicated in cancer progression. This pathway involves the stabilization and nuclear translocation of β‐catenin, which then activates the transcription of Wnt‐responsive genes. H19 is associated with miRNAs for the regulation of the EMT pathway, resulting in alteration of cell growth [70]. MALAT1 promotes the nuclear translocation of β‐catenin, enhancing Wnt signaling and contributing to tumor aggressiveness in HNSCC [71]. MALAT1 also promotes resistance to cisplatin in OSCC by activating the PI3K/AKT/m‐TOR pathway [72]. Other lncRNAs, such as MEG3, UCA1, TUG1, NEF, and LINC00473, regulate Wnt/β‐catenin signaling, influencing β‐catenin expression, cell proliferation, apoptosis, and radio‐resistance in HNSCC cells [73, 74, 75, 76]. Association of m6A/HOXA10‐AS/ITGA6 affects oxidative stress responses and activates the Notch signaling pathway, which drives tumor progression in LSCC [77]. The JAK/STAT3 signaling pathway plays a pivotal role in cancer progression, frequently activated in HNSCC. The STAT3 pathway regulates EMT and self‐renewal of CSCs, allowing the transmission of signals from the cell membrane to the nucleus. FOXD2‐AS1 enhances STAT3 activity by acting as a scaffold that brings STAT3 and PRMT5 (protein arginine N‐methyltransferase 5) into close proximity, leading to increased STAT3 transcriptional activity [65]. This interaction promotes CSC‐like properties and contributes to chemotherapy resistance in HNSCC. Conversely, lincRNA‐p21 functions as a tumor suppressor by inhibiting JAK2/STAT3 signaling [66]. It directly binds to STAT3, preventing its activation, and induces G1 cell cycle arrest and apoptosis in cancer cells [66, 78]. Activated STAT3 can also contribute to epigenetic changes that promote oncogenesis. For example, STAT3 binds to the promoter region of the HOTAIR gene, enhancing its expression and thereby promoting EZH2‐mediated silencing of tumor suppressor genes in HNSCC [79].

The TGF‐β/Smad pathway plays a complex role in HNSCC. While TGF‐β has tumor‐suppressive roles in early cancer stages, it often promotes tumor progression and EMT in advanced stages of HNSCC [80]. TGF‐β/Smad signaling in association with lncRNA ANRIL or EPB41L4A‐AS2 regulates cell growth and migration in HNSCC [81, 82]. LINC01133 interacts with TGF‐β/Smad signaling and promotes the expression of genes that drive EMT and metastasis [83]. LncRNA UCA1 is shown to promote EMT and metastasis through inducing TGF‐β through JAG1/Notch signaling in a lncRNA‐miRNA‐mRNA regulation mechanism [84]. These findings highlight the interactions between the various oncogenic signaling pathways and lncRNA, which suggests their potential as therapeutic targets for cancer treatment.

Several areas on lncRNAs research remain underexplored. For instance, the functional variability of lncRNAs across different anatomical subtypes of HNSCC, such as oral, laryngeal, and hypopharyngeal cancers, remains unclear. Additionally, the role of lncRNAs in metabolic reprogramming, which involves altering the energy production processes of cancer cells to support their rapid growth, needs further investigation. Understanding these aspects could yield new therapeutic targets, especially for overcoming resistance to current treatments. Furthermore, more studies are needed to explore how lncRNAs carried in exosomes (small vesicles released by cells) facilitate communication between HNSCC cells and other cells in the tumor microenvironment, which could provide insights into metastasis and immune evasion mechanisms.

## Highlights for well‐studied lncRNAs as an oncogene or tumor suppressor gene in HNSCC

Studies have characterized lncRNA expression profiles in HNSCC tissues, revealing distinct patterns compared to normal tissues. A study identified 488 differentially expressed lncRNAs in HNSCC using The Cancer Genome Atlas (TCGA) database [85]. To further confirm the expression of 24 chosen lncRNAs, they examined the expression levels in clinical tissues and found four significant lncRNA biomarkers that were highly expressed in HNSCC tissues (LINC00460, LINC00941, CTC‐241F20.4, and RP11‐357H14.17) with two of these lncRNAs (LINC00460 and CTC‐241F20.4) being elevated in late‐stage HNSCC and HPV‐negative statuses [85]. The prevalence and expression patterns of lncRNAs in HNSCC have implications for diagnosis, prognosis, and therapy. Dysregulated lncRNAs in HNSCC hold promise as biomarkers for early detection and monitoring, while targeting these non‐coding RNAs may provide innovative therapeutic strategies for improving treatment outcomes. In this review, we discussed some well‐studied lncRNAs involved in HNSCC. Table 1 lists the functional role and signaling pathways of specific lncRNAs in HNSCC.

**Table 1:** List of lncRNAs in promotion and suppression of HNSCC.

LncRNA	Genome	Expression	Functional role and pathways involved	References
*ELDR*	7p11.2	• Upregulated in OSCC	• Promoted cell growth through interaction with ILF3 and CTCF • Accelerated G2/M phase via stabilization of CTCF, resulting in FOXM1‐AURKA‐mediated G2/M progression	[86, 87]
*MALAT1*	11q13.1	• Overexpressed in HNSCC tissue and plasma patient samples • Upregulated in ESCC samples	• Contributed to G2/M phase arrest, promotion of apoptosis, and cell migration and invasion • Affected cell invasion, viability, and migration of EMT of ESCC cells through regulation of EZH2‐Notch1 signaling pathway • Dephosphorylation of ATM‐CHK2 pathway, leading to promotion of ESCC growth	[90, 91, 92, 93, 126]
*NEAT1*	11q13.1	• Overexpressed in LSCC and OSCC samples • Upregulated in SNSCC samples	• Promoted MMP11 transcription through heat shock factor 1 binding and LSCC progression via binding miR‐340‐5p • Activation of PI3K/AKT pathway via downregulation of miR‐195‐5p • Promoted LSCC proliferation and tumorigenesis through Wnt signaling pathway activation via the miR‐411‐3p/FZD3 ceRNA axis • NEAT1_2 as a ceRNA to regulate ATAD2 expression by via sponging miR‐106b‐5p	[76, 99, 100, 101, 102, 103]
*HOTAIR*	12q13.13	• Upregulated in HNSCC, LSCC, and OSCC samples	• Feed‐forward regulatory loop interaction with HuR via sponging miR‐7 • Promoted LSCC progression by inducing M2 polarization via PI3K/p‐AKT/AKT pathway	[105, 106]
*HOTTIP*	7p15.2	• Upregulated in HNSCC, OSCC, NPC, OTSCC	• *HOTTIP* and M1 exosomes activated TLR5/NF‐κB signaling pathway via sponging miR‐19a‐3p and miR‐19b‐3p • *HOTTIP* silencing induced apoptosis and inhibited cell proliferation, invasion, and metastasis in NPC cells	[108, 109, 110, 127]
*UCA1*	19p13.12	• Overexpressed in LSCC, OSCC, ESCC, and HSCC samples • Upregulated in OSCC‐CSC‐sEV samples	• Promoted LSCC cell proliferation, invasion, and migration through activation of Wnt/β‐catenin signaling pathway • Activated PI3K/p‐AKT/AKT pathway through binding of miR‐134 in CSC‐secreted sEVs • Induced TGF‐β through JAG/Notch1 signaling pathway via binding miR‐124	[81, 84, 111, 112, 113, 114, 115, 116]
*PCAT‐1*	8q24.21	• Overexpressed in HNSCC samples • Upregulated in LC	• Positively correlated with AKT1 and c‐Myc expression in HNSCC, and depletion led to activation of p38 MAPK signaling and Caspase 9 mediated apoptosis • Promoted cell proliferation and metastasis in LC patients via sponging miR‐210‐3p	[118, 128]
*PVT1*	8q24.21	• Overexpressed in HNSCC, LSCC, and OSCC samples	• Promoted metastasis in HNSCC • Inhibited HNSCC stemness through interaction with YAP1 via sponging miR‐375 • PVT1 knockdown inhibited TGF‐β expression	[43, 129, 130, 131, 132]
*H19*	11p15.5	• Upregulated in LSCC and TSCC samples	• Promoted metastasis in LSCC through binding of miR‐148a‐3p and subsequent binding of DNMT1, inducing DNA methylation	[67, 70]
*MEG3*	14q32. 3	• Downregulated in HNSCC, OSCC, and NPC samples	• Regulated EMT process in HNSCC via sponging miR‐421 • Mediated expression level of SQSTM1 and promoted NPC cell migration and invasion • Modulated JAK–STAT pathway in OSCC via sponging miR‐548d‐5p	[133, 134, 135]
*TUG1*	22q12.2	• Overexpressed in LSCC samples and correlated with lymph node metastasis • Upregulated in OSCC and TSCC samples	• Promoted proliferation in LSCC cells via inhibition of apoptosis and acceleration of cell division • Induced apoptosis and suppressed cell proliferation through knockdown of *TUG1* via Wnt/β‐catenin signaling pathway • Regulated OSCC cell proliferation and invasion, MAPK signaling pathway, and EMT process via miR‐593‐3p	[73, 136, 137, 138]
*SNHG1*	11q12.3	• Overexpressed in OSCC • Upregulated in NPC	• Promoted TSCC cell migration, invasion and EMT process by upregulating MTFR1 • Mediated NPC proliferation through AKT signaling pathway and induced EMT process via sponging miR‐145‐5p	[139, 140]

### ELDR

EGFR long non‐coding downstream RNA (ELDR) is a relatively new human lncRNA and is adjacent to the EGFR gene on chromosome 7 on the opposite strand. The murine ELDR gene has a human homolog that is present on chromosome 11, and its function is yet to be determined. We have shown that ELDR is an oncogene for oral cancer and interacts with ILF3 and CTCF for promotion of cell growth [86]. A subsequent study showed that exogenous ELDR increased proliferation in normal oral keratinocytes by accelerating the G_2_/M phase of cell cycle progression through stabilization of CTCF, resulting in FOXM1‐AURKA‐mediated G_2_/M progression [87]. In a therapeutic aspect, intratumor delivery of ELDR significantly shrinks tumor growth in patient‐derived xenograft models [86, 88]. However, the therapeutic effects of ELDR should be evaluated in different preclinical models in the presence of an intact immune system.

### MALAT1

Metastasis‐associated lung adenocarcinoma transcript 1 (MALAT1) (chromosome 11q13.1) is a well‐known lncRNA that was first discovered in early‐stage non‐small cell lung cancer (NSCLC) cell lines and patient samples [89]. MALAT1 is overexpressed in HNSCC cell lines and tissues compared to adjacent non‐tumorous samples [90, 91, 92]. The knockdown of MALAT1 leads to aggregation of cells at the G_2_/M phase, which causes the promotion of apoptosis, inhibition of cell migration and invasion, and reduction of colony formation. Overexpression of MALAT1 leads to the dephosphorylation of the ATM‐CHK2 pathway, an essential pathway that is involved in DNA damage response and G_2_/M arrest, thereby promoting ESCC growth [93]. MALAT1 acts as a ceRNA to sponge miRNAs, such as miR‐101‐3p, miR‐217, miR‐30a, and miR‐125b in HNSCC [54, 92, 94]. Co‐treatment of MALAT1 with cisplatin and radiation enhances the therapeutic efficacy in the HNSCC preclinical model [91].

### NEAT1

Nuclear‐enriched abundant transcript 1 (NEAT1) is on chromosome 11q13.1. The architectural role of NEAT1 in paraspeckle formation—dynamic nuclear bodies with numerous roles in gene expression—is well‐researched [95]. Recent studies have observed two isoforms of NEAT1, the shorter isoform *NEAT1_1* (3.7 kb in human) and the longer isoform *NEAT1_2* (22.7 kb in human) [96], which each display different roles in regulating the different phenotypes in cancer cells [97, 98]. While *NEAT1_2* may function in the formation and assembly of paraspeckle, *NEAT1_1* is found to have paraspeckle‐independent functions [96]. Although isoform‐specific contributions are still unknown, studying both could be effective in determining the role of NEAT1 as a tumor promoter or tumor suppressor in cancer. Regarding research on HNSCC, NEAT1 is overexpressed in LSCC [99, 100, 101], OSCC [102] and sinonasal squamous cell carcinoma [103] when compared to adjacent non‐cancerous tissues. In LSCC cells, knockdown of NEAT1 prevents cell proliferation and invasion while inducing cell cycle arrest at the G1 phase and promoting apoptosis [99]. In a xenograft model, knockdown of NEAT1 reduces tumor growth and induces cancer cell apoptosis. NEAT1 may also be involved in the activation of the Wnt signaling pathway by acting as a ceRNA to bind miR‐411‐3p and thus promoting FZD3 expression [101].

### HOTAIR

Homeobox (HOX) Transcript Antisense Intergenic RNA (HOTAIR) (chromosome 12q13.13) is highly expressed in cancer tissues and plays a role in tumor progression, invasion, and metastasis [104]. HOTAIR expression is also high in HNSCC [104, 105, 106, 107]. Mechanistically, HOTAIR regulates transcription by binding histone modification complexes, PRC2 and Lysine‐Specific Demethylase 1 (LSD1), to the HOXD locus on chromosome 2, which then alters chromatin states by coupling histone H3K27 methylation and H3K4 demethylation, and this leads to metastasis. In OSCC, silencing HOTAIR led to decreased binding of EZH2 and H3K27me3 with the E‐cadherin promoter, suggesting that HOTAIR may repress the expression of E‐cadherin by recruiting EZH2 and, thereby, inhibiting cancer cell proliferation and invasion [104]. Another study found that the knockdown of HOTAIR by shRNA in HNSCC cell lines inhibited cell proliferation and led to cell cycle arrest and apoptosis [105]. A possible feed‐forward regulatory loop between HuR and HOTAIR supports the theory that HuR activity is repressed by miR‐7, and HOTAIR alleviates this by acting as a sponge for miR‐7 in HNSCC [105]. A recent study on LSCC observed the potential role of HOTAIR in activating the PI3K/p‐AKT/AKT pathway for induction of the polarization of macrophages to M2 phenotype [106]. These studies have highlighted the potential of HOTAIR as a biomarker for HNSCC.

### HOTTIP

HOXA transcript at the distal tip (HOTTIP) (chromosome 7p15.2) is located at the 5′ end of the homeobox A (HOXA) cluster and manages the activation of numerous 5′ HOXA genes [108]. HOTTIP engages in a multitude of cellular processes, such as cell growth, invasion, migration, and apoptosis. Recent reports suggested that HOTTIP is upregulated in HNSCC specimens [108, 109]. HOTTIP is a key molecule in exosomes derived from M1 macrophage and activation of the TLR5/NF‐κB signaling pathway through competitively sponging miR‐19a‐3p and miR‐19b‐3p [110]. In another study in nasopharyngeal carcinoma cells (NPC), HOXA13 was downregulated due to knockdown of HOTTIP, and this subsequently led to the suppression of cell proliferation, invasion, and metastasis, and induced apoptosis [108]. Thus, examining the role of HOTTIP as a potential therapeutic target could be crucial in the clinical evaluation of cancer.

### UCA1

Urothelial carcinoma associated 1 (UCA1) (chromosome 19p13.12) was first discovered in 2006 in human bladder cancer [111]. UCA1 is also involved in nasopharyngeal carcinoma tumorigenesis and metastasis [81]. Further, compared to adjacent non‐tumor tissue, patient tumor samples with LSCC [112], OSCC [102], ESCC [113], and hypopharyngeal squamous cell carcinoma [114] display higher expression of UCA1. Several studies reported that the role of UCA1 in other malignant tumors and specifically its function in neoplasm, apoptosis, anti‐cancer drug resistance, metabolism, and crosstalk with miRNAs [81, 115, 116]. Overexpression of UCA1 contributes to the activation of the Wnt/β‐catenin signaling pathway by inhibiting the negative activator of this signaling pathway while promoting the expression of β‐catenin [112]. In another study, Wu *et al*. analyzed the potential role of UCA1 in the PI3K/AKT pathway through binding of miR‐134 and OSCC cancer stem cell‐derived small extracellular vesicle (CSC‐derived sEV) which led to the promotion of M2 polarization of macrophages [117].

### PCAT‐1

Prostate cancer‐associated transcript 1 (PCAT‐1) is located on chromosome 8q24.21, approximately 725 kb upstream of the Myc oncogene, and the region is frequently amplified in HNSCC. In fact, overexpression of PCAT‐1 in HNSCC patient samples compared to adjacent non‐tumor tissues was also noted [118]. c‐Myc and AKT1 positively correlate with PCAT‐1 expression in HNSCC. We showed that depletion of PCAT‐1 inhibits HNSCC cell growth, c‐Myc and AKT1 expression, and induces apoptosis [118]. Targeted inhibition of PCAT‐1 reduces tumor growth in a preclinical model.

## Clinical implications and future directions

The aberrant expression of lncRNAs is closely associated with several pathophysiological functions that stimulate tumorigenesis. Consequently, lncRNAs hold a great promise for therapeutic applications for cancers including HNSCC. Targeted inhibition or restoration of lncRNAs can alter the cancer cells' regulatory networks in a context‐dependent manner. The lncRNAs can simultaneously regulate multiple targets, which can be beneficial or challenging due to complicated feedback mechanisms. Therefore, understanding the detailed mechanism and function of lncRNA in HNSCC is extremely important, especially in identifying it as a therapeutic target.

As discussed above, deregulation of lncRNAs, such as ELDR, HOTAIR, LEMD1‐AS1, and MALAT1, has been associated with the onset and progression of HNSCC [86, 119]. For example, ELDR is highly expressed in OSCC patient samples and in cell lines [86], and ectopic expression of ELDR in normal oral keratinocytes enhances cell growth [87]. MALAT1 is upregulated in OSCC and is correlated with differentiation and clinical stage [120]. HOTAIR is highly expressed in OSCC and the level of expression is correlated with tumor lymph node metastasis stage, histological grade, and regional lymph node metastasis [121]. LncRNA LEMD1‐AS1 is elevated in OSCC [122]. So far, there are 35 clinical trials using lncRNAs, and five of them are with HNSCC. The relevant clinical trials have been summarized in Table 2 and can be accessed on the following website: https://clinicaltrials.gov/.

**Table 2:** List of lncRNAs in HNSCC clinical trials.

ID number	LncRNA	General description
NCT03469544	*HOTAIR*	*HOTAIR* as a possible biomarker in addition to midkine, a heparin‐binding growth factor related to tumor stage progression
NCT04946968	*EGFR‐AS1*	Evaluating the efficacy of oral Dacomitinib for patients with Epidermal Growth Factor Receptor (EGFR)‐driven advanced solid tumors with low *EGFR‐AS1* expression or other novel biomarkers
NCT05708209	*MALAT1*	*MALAT1* as a potential salivary biomarker in OSCC through miRNA‐124 interaction
NCT05730855	*DQ786243*	*DQ786243* as a possible salivary diagnostic marker of oral potentially malignant lesions through miRNA‐146a interaction
NCT05821179	*LINC00657*	Salivary *LINC00657* and miRNA‐106a as possible diagnostic markers in OSCC

Based on available clinical trial databases, five clinical trials on lncRNAs as potential biomarkers in HNSCC have been studied. A clinical trial analyzed the expression levels of HOTAIR and expression levels of midkine, a heparin‐binding growth factor related to tumor stage progression, in liquid biopsy of patients and healthy individuals to determine the potential of HOTAIR and midkine as biomarkers in thyroid cancer (NCT03469544). Another completed clinical trial in this field studied the use of salivary lncRNA LINC00657 and miRNA‐106a as diagnostic biomarkers in OSCC and the chronic inflammatory condition oral lichen planus (NCT05821179). This study recruited 36 subjects and divided them into three equal groups: those with OSCC, those with oral lichen planus, and those without either condition. LINC00657 had a statistically significant higher expression in the OSCC group compared to the other two groups, and LINC00657 had higher diagnostic accuracy than miRNA‐106a [123]. An observational study on MALAT1 as a potential salivary biomarker in OSCC through miRNA‐124 interaction has been completed (NCT05708209). This study recruited 40 subjects, with half of the group consisting of patients with OSCC and the other half consisting of healthy subjects. The study found a statistically significant increase in the expression of the lncRNA MALAT1 in OSCC patients compared to the control. Furthermore, the results showed statistically significant increases in MALAT1 expression in patients with metastatic cancer with lymph node involvement compared to non‐metastatic cancer [124]. Another clinical trial is underway, observing the efficacy of the oral drug, Dacomitinib, for patients with Epidermal Growth Factor Receptor (EGFR)‐driven advanced solid tumors with low EGFR‐AS1 expression (NCT04946968). This trial is currently in Phase II with 104 participants enrolled.

An additional study evaluated lncRNA DQ786243 as a potential biomarker and its effect on miRNA‐146a (NCT05730855). The expression level of DQ786243 and miRNA‐146a were observed in the saliva of three different groups: patients suffering from oral lichen planus, patients suffering from leukoplakia, and healthy individuals that did not suffer from any oral mucosal lesions. Results of this study have not been published yet, but assessing DQ786243 as a potential biomarker could be useful in diagnosing oral malignant lesions. Overall, these clinical trials highlight the growing and promising impact of lncRNAs in the care of patients with HNSCC, namely in the diagnosis and prognosis of the condition. Although the diagnosis of head and neck squamous cell carcinoma is not difficult to make, having a signature of lncRNAs may be useful as prognostic biomarkers. It may also be helpful as predictive biomarkers in areas where HNSCC is highly prevalent. Therefore, it may be reasonable to identify a group of lncRNAs as a predictive and/or prognostic biomarker for HNSCC.

Identifying lncRNAs as a potential biomarker for HNSCC would be beneficial; however, targeting lncRNA faces several challenges. In preclinical models, intratumor delivery of lncRNAs shows promising results, but clinical applications targeting lncRNAs are hindered by delivery, stability, and specificity. Maintaining the stability of lncRNAs *in vivo* is difficult, and currently, in clinical settings, there is no effective lncRNA delivery system in tumor cells. Additionally, problems regarding lncRNA specificity include undesired on‐ and off‐target effects where a therapeutic intervention aimed at a certain lncRNA inadvertently affects other pathways or cellular processes, and this leads to unwanted side effects or complications [125]. Due to these challenges, investigations are needed in chemical technology, focusing on improving the stability of lncRNA *in vivo*, and nanotechnology, which addresses the need to find an efficient delivery vehicle for lncRNAs *in vivo* or targeted delivery with viral‐based vectors. Another crucial point to mention is the inconsistency of results between studies. Reproduced outcomes are essential, and these varying results may be questioned due to a lack of consensus. Therefore, there is a need to address these variabilities and overcome the challenges of targeted therapy that uses lncRNA in order to improve its efficacy in clinical settings.

In conclusion, lncRNAs are promising targets for cancer therapeutics and have immense potential to serve as biomarkers for cancer prediction and prognosis, which will steer decision‐making clinically. The lncRNA research is an emerging field with great promise for cancer treatment, especially to target ‘undruggable’ genes. Further investigation is indeed necessary to overcome and address these challenges and translate into clinical application. Therefore, better understanding of the regulation of lncRNAs will be a stepping‐stone for future therapeutic modalities for HNSCC.

## Conflict of interest

The authors declare no conflict of interest.

## Author contributions

RBR conceived the project and procured the funding. All authors (ETT, RAP, AC, and RBR) contributed to the writing of this review and approved it.
